# Shedding light on the non-operative treatment of the forgotten side of the knee: rehabilitation of medial collateral ligament injuries—a systematic review

**DOI:** 10.1136/bmjsem-2023-001750

**Published:** 2024-06-25

**Authors:** Jasmine Svantesson, Ramana Piussi, Elin Weissglas, Eleonor Svantesson, Alexandra Horvath, Erik Börjesson, Andy Williams, Robert Prill, Kristian Samuelsson, Eric Hamrin Senorski

**Affiliations:** 1 Sportrehab, Gothenburg, Sweden; 2 Department of Health and Rehabilitation, University of Gothenburg, Institute of Neuroscience and Physiology, Goteborg, Sweden; 3 Sahlgrenska Sports Medicine Center, Gothenburg, Sweden; 4 Department of Orthopedics, University of Gothenburg Sahlgrenska Academy, Goteborg, Sweden; 5 Department of Internal Medicine and Clinical Nutrition, Institute of Medicine, Sahlgrenska Academy, University of Gothenburg, Gothenburg, Sweden; 6 FIFA Medical Centre of Excellence, Fortius Clinic City, London, Greater London, UK; 7 Center of Orthopaedics and Traumatology, Brandenburg Medical School Theodor Fontane, Brandenburg an der Havel, Germany; 8 Faculty of Health Sciences, Brandenburg Medical School Theodor Fontane, Brandenburg an der Havel, Germany; 9 Department of Orthopaedics, Institute of Clinical Sciences, Gothenburg, Sweden; 10 Department of Orthopaedics, Institute of Clinical Sciences, Sahlgrenska Academy, University of Gothenburg, Gothenburg, Sweden; 11 Swedish Olympic Comitee, Stockholm, Sweden

**Keywords:** Knee, Rehabilitation, Injury

## Abstract

**Objective:**

The purpose of this study was to review the current literature regarding the non-operative treatment of isolated medial collateral ligament (MCL) injuries.

**Design:**

Systematic review, registered in the Open Science Framework (https://doi.org/10.17605/OSF.IO/E9CP4).

**Data sources:**

The Embase, MEDLINE and PEDro databases were searched; last search was performed on December 2023.

**Eligibility criteria:**

Peer-reviewed original reports from studies that included information about individuals who sustained an isolated MCL injury with non-surgical treatment as an intervention, or reports comparing surgical with non-surgical treatment were eligible for inclusion. Included reports were synthesised qualitatively. Risk of bias was assessed with the Risk of Bias Assessment tool for Non-randomized Studies. Certainty of evidence was determined using the Grading of Recommendations Assessment Development and Evaluation.

**Results:**

A total of 26 reports (1912 patients) were included, of which 18 were published before the year 2000 and 8 after. No differences in non-operative treatment were reported between grade I and II injuries, where immediate weight bearing and ambulation were tolerated, and rehabilitation comprised different types of strengthening exercises with poorly reported details. Some reports used immobilisation with a brace as a treatment method, while others did not use any equipment. The use of a brace and duration of use was inconsistently reported.

**Conclusion:**

There is substantial heterogeneity and lack of detail regarding the non-operative treatment of isolated MCL injuries. This should prompt researchers and clinicians to produce high-quality evidence studies on the promising non-operative treatment of isolated MCL injuries to aid in decision-making and guide rehabilitation after MCL injury.

**Level of evidence:**

Level I, systematic review.

**WHAT IS ALREADY KNOWN**

- Medial collateral ligament (MCL) injuries are prevalent among knee ligament injuries, and commonly non-surgical treatment is the primary approach for isolated MCL injuries.

**WHAT ARE THE NEW FINDINGS**

- There were no reported discernible differences in treatment and outcomes between grade I and II MCL injuries, suggesting that similar management strategies are used.
- Immediate weight bearing and ambulation, when tolerated, are feasible practices following MCL injury, potentially facilitating quicker recovery.
- The use of braces in MCL injury management varies widely across studies, indicating a lack of consensus on their effectiveness or necessity in treatment protocols.
- Despite advancements, there is currently very low-quality evidence for the non-operative treatment of MCL injuries, and significant knowledge gaps persist, emphasising the need for further research and standardisation of treatment approaches.

## Introduction

The knee joint has several ligaments that act as passive stabilisers and help counteract forces acting on the knee joint.^1^ On the medial side of the knee joint, the gastrocnemius muscle, medial hamstring muscles and the medial collateral ligament (MCL) act as important stabilisers for valgus forces. The MCL is 8 cm to 10 cm long and originates proximally and centrally on the medial femoral epicondyle.^2^ It attaches distally posterior to the medial condyle of the tibia and the pes anserinus, approximately 5 cm to 7 cm distal to the joint line.^3 4^ The ligament is divided into the superficial MCL and the deep MCL. The deep portion of the MCL can also be divided into two components: the meniscofemoral and the meniscotibial components. Both components attach to the medial meniscus.^3 4^ Synergistically to the MCL, the posterior oblique ligament is the predominant ligamentous structure on the posterior medial corner of the knee joint. It acts as a stabiliser of internal rotation close to extension in the knee joint.^5^ The deep layer of the MCL can be regarded as a thickening of the joint capsule.^6^

Injuries to the MCL are the most common ligament injury to the knee.^7^ An elite male football team can expect an average of two MCL injuries in the team during one season, which can be compared with one anterior cruciate ligament injury over two seasons.^8^ The MCL is often injured in connection with trauma during sports activities.^3^ The injury usually occurs when the knee is subjected to valgus stress at 20^°^ of knee flexion due, for example, to a blow from the lateral side of the leg or knee, and it frequently occurs during the simultaneous external rotation of the tibia.^9 10^ According to the Fetto and Marshall^11^ classification, there are three grades of MCL injury: I (no valgus laxity), grade II (valgus laxity at 30° of flexion) and grade III (valgus laxity at 0° and 30° of flexion),^11 12^ with increasing medial instability from grade I to III, where grade I represents 3–5 millimetres (mm) of medial joint space opening; grade II represents 6–10 mm of medial joint space opening, while grade III represents >10 mm of medial joint space opening.^13^

Isolated MCL injuries are primarily treated non-surgically, including physiotherapeutic intervention in rehabilitation and exercise therapy.^9^ The non-surgical treatment of most isolated injuries is logical, as the deep part of the MCL has historically been described as having good self-healing capacity because it is related to the joint capsule.^14^ Recent advances in clinical practice have suggested that the location of the MCL injury better explains why some MCL injuries heal well. In contrast, femoral MCL injuries tend to do well unless there is an avulsion off the bone, while tibial injuries can be more problematic. As part of the non-surgical treatment of isolated MCL injuries, typically grade II or III, a hinged brace can protect the knee from further valgus stress.^9^ Some injuries may, however, be recommended for acute surgery: grade III isolated MCL tears in athletes, with medial joint opening in full extension with valgus applied, or a tibial avulsion with the MCL folded into the medial joint, or a Stener lesion with the MCL lying superficial to the pes anserine tendons; or a positive dial test indicating anteromedial rotatory instability. Following the failure of non-surgical treatment, a patient may experience persistent symptoms and/or activity limitations, and surgical treatment may be considered.^13^ Despite the practice of non-surgical treatment for most isolated MCL injuries, the evidence regarding the structure and content of rehabilitation is limited. In addition, there is no aggregated evidence on the outcome of non-surgical treatment after MCL injury. Therefore, this study systematically reviewed the current literature regarding the non-surgical treatment of isolated MCL injuries. A secondary objective was to investigate surgical and non-surgical treatment outcomes for MCL injuries.

## Method

### Patient and public involvement statement

Neither patients nor the public were involved at any stage during the planning or execution of this study.

The review was conducted according to the ‘Preferred Reporting Items for Systematic Reviews and Meta-Analyses’ (PRISMA)^15^ and was registered at the Open Science Framework website (https://doi.org/10.17605/OSF.IO/E9CP4). General recommendations for the production of systematic reviews have been followed.^16^

### Eligibility criteria

All the reports from original studies written in English and Swedish that included information about individuals who sustained an isolated MCL injury with non-surgical treatment as an intervention or reports of studies comparing surgical with non-surgical treatment for isolated MCL injuries were eligible for inclusion. Reports from clinical studies of humans on the treatment type or outcomes were eligible for inclusion. Reports from studies involving animals or cadavers, case reports, editorials, expert opinions, narrative reviews, systematic reviews, meta-analyses, abstracts, book chapters and opinion pieces were excluded. When reports that could potentially be included did not report the outcome of interest, the corresponding authors were contacted for additional data. Reports were excluded if no response was received from a contacted author. The latest published report was included for studies publishing data from the same individuals or cohort several times.

### Information sources

Four literature searches were performed by an experienced librarian at the Sahlgrenska University Hospital library on 11 November 2020, 22 December 2021, 18 November 2022 and 20 December 2023, respectively. The Excerpta Medica dataBASE (Embase), Medical Literature Analysis and Retrieval System Online (MEDLINE) and Physiotherapy Evidence Database (PEDro) databases were searched to identify eligible reports.

### Search strategy

The search comprised terms such as “Medial Collateral Ligament” specified to the knee and synonyms to gather all the published reports on MCL. Search words used were: Medial Collateral Ligament, Knee[mesh] OR knee medial collateral ligament[tiab] OR tibial collateral ligament[tiab] OR Ligamentum Collaterale Tibiales[tiab] OR Medial collateral ligament of the knee[tiab]. For the complete search strategy, please see online supplemental tables 1–3. Systematic reviews and meta-analyses published on this topic were screened for additional reports.

10.1136/bmjsem-2023-001750.supp1 Supplementary data

### Selection process

Results from the search were uploaded into the Rayyan QCRI^17^ web application, where all duplicates were removed. Two authors (JS and RP) performed the selection process independently. The two authors separately screened titles and abstracts of the records from the electronic searches to identify potentially eligible reports. No automated or semiautomated approaches were used. Authors of primary reports were not contacted to clarify eligibility. The senior author (EHS) was consulted in the event of disagreement. In the event of an abstract not containing information that could inform decision-making for inclusion or exclusion, these reports were included in the full-text assessment. A Cohen’s kappa coefficient was calculated, showing moderate agreement (K=0.42).^18^

### Data collection process

The two authors (JS and RP) responsible for the data collection process developed a data extraction sheet and pilot tested it on two included reports. The extraction sheet was then sent to four authors (AH, ES, KS, EHS) for any adjustment. Two authors (JS and RP) extracted data independently to a Microsoft Excel spreadsheet (V.16; Microsoft Corporation, Redmond, Washington). After the extraction, the responsible authors (JS and RP) checked each other’s extraction sheets. That is, the extraction was performed again to validate the data extracted. Any disagreement was resolved with a discussion with the senior author (EHS).

### Data items

In the present systematic review, we used an explorative and data-driven approach. The primary outcome of interest was the type of treatment (eg, exercise therapy or bracing) for isolated MCL injury. Furthermore, we aimed to explore the outcomes of the non-surgical treatment of isolated MCL injuries. The treatment outcome was defined as any reported outcome, such as strength, the answer to a patient-reported outcome, time to return to sport, clinical laxity or MRI findings in patients treated non-surgically after an MCL injury. The secondary outcome was the comparison of outcomes between the surgical and non-surgical treatment of MCL injuries. The data were extracted according to the following headings: authors; year; journal; study type; type of injury; study purpose; total study size; follow-up time; dropout rate; sex; age; type of treatment; grading of injury; previous activity level; patient-reported outcomes and return to sports. Data were extracted from tables, figures or text, depending on where it was presented in the included report. For multiple reports from the same study, data from each report were extracted separately and then combined in tables (see footnote under table 1).

**Table 1:** Details of included reports

Lead author	Year	Study type	Patients (n)	Age (year)	Non-surgery/surgery (n)	Follow-up (M)	Injury grade		
							I	II	III
Ballmer and Jakob^27^	1988	Prospective	23	n.r.	23/0	18	0	0	23
Derscheid and Garrick^31^	1981	Prospective	51	n.r.	51/0	n.r.	23	28	0
Ellsasser *et al* 25	1974	Retrospective	75	n.r.	52/23	n.r.	n.r.	n.r.	n.r.
Fetto and Marshall^11^	1978	Cohort	263	13–78	115/150	6–144	8/0	60	28
Halkjear-Kristensen and Ingemann-Hansen^39 40^ *	1985	Prospective	84	n.r.	23/61	n.r.	n.r.	n.r.	n.r.
Holden *et al* 28	1983	Prospective	89	n.r.	51/38	n.r.	17	51	0
Indelicato^32^	1983	Prospective	36	19.4	24/27	30	0	0	36
Indelicato *et al* 33	1990	Prospective	28	n.r.	28/0	46	0	0	28
Jang and Kim^26^	2023	Retrospective	50	26.8	50/0	12	0	0	50
Jones *et al* 34	2009	Case series	34	37	34/0	20.4	n.r.	n.r.	n.r.
Jones *et al* 35	1986	Prospective	24	16.5	24/0	6	0	0	22
Kannus^41 42^ *	1988/9	Cross-sectional	25	35±12	25/0	n.a.	0	0	25
Kannus^43^	1988	Prospective	96	36±15	81/0	96	0	54	27
Logan *et al* 45	2018	Retrospective	301	n.r.	323/14	n.r.	n.r.	n.r.	n.r.
Lundberg and Messner 29	1996	Prospective	38	24	38/0	120	16	22	0
Lundberg and Messner^48^	1997	Matched cohort	40	24	20/0	120	n.r.	n.r.	n.r.
Lundblad *et al* 44	2013	Retrospective	346	n.r.	n.r.	132	n.r.	n.r.	n.r.
Lundblad *et al* 8	2019	Prospective	115	n.r.	128/2	3 seasons	74	47	5
Motamedi *et al* 30	2017	Retrospective	47	n.r.	n.r.	n.r.	46		1
Petermann *et al* 36	1993	Cohort	86	30.2	86/0	44.2	39	41	6
Reider *et al* 37	1994	Cohort	35	20.1	35/0	60	n.r.	n.r.	n.r.
Sandberg *et al* 38	1987	Prospective	43	n.r.	24/19	13	n.r.	n.r.	n.r.
Yagishita *et al* 46	2019	Comparative	32	27.2±3	n.r.	1	0	32	0
Zou *et al* 47	2020	Cohort study	52	36.5±5	n.r.	6	n.r.	n.r.	n.r.

*For the two reports by Kannus^41 42^ and Halkjaer and Ingemann-Hansen,^39 40^ different outcomes for the same cohort were reported in different publications. As a result, both reports are included, but the number of patients was counted once.

M, months; n, number; n.a., not applicable; n.r., not reported.

### Report risk of bias assessment

The risk of bias^19^ for the included reports was assessed with the Risk of Bias Assessment tool for Non-randomized Studies (RoBANS). The RoBANS contains six domains, including the selection of participants, confounding variables, measurement of intervention (exposure), blinding of outcome assessment, incomplete outcome data and selective outcome reporting.^20^ For each included domain, the risk of bias is judged as high, low or unclear. The RoBANS is not a scale but a domain-based evaluation tool compatible with the GRADE approach.^20^ The reports were independently graded by two authors (JS and RP), and any disagreements were resolved by discussion. The domains ‘incomplete outcome data’ and ‘selective outcomes reporting’ were qualitatively assessed to explore the possible risk of publication bias.

### Critical appraisal

The included reports were assessed using the Methodological Index for Non-Randomised Studies (MINORS).^21^ All the included reports were independently graded by two authors (JS and RP). Disagreements were resolved by consensus. For non-comparative reports, eight items were assessed; for comparative reports, there were four additional items, resulting in 12 items. All the items were graded with a score of 0–2 points, with 0 representing an item not reported, 1 representing an item reported inadequately, and 2 representing an item reported adequately. As a result, the maximum score was 16 points for the non-comparative reports and 24 points for the comparative reports, respectively. We interpreted the scores as follows: for non-comparative reports, 0–4 very low quality; 5–8 low quality; 9–12 fair quality and 13–16 high quality. For comparative reports, 0–6 are very low quality, 7–12 are low quality, 13–18 are fair quality and 19–24 are high quality.^21^

### Synthesis method

Data synthesis was performed according to the Emerging Evidence Syntheses.^22^ The authors synthesised the literature qualitatively, in text and divided it into subject areas based on different data types. To synthesise the results and type of non-surgical treatment after isolated MCL injuries, the authors organised results according to headings comprising reported treatment options in the included reports: exercise therapy, bracing and other treatment. Finally, a heading was created to compare the surgical and non-surgical treatment results of MCL injuries. Whenever possible, quantitative data were summarised in tables. Due to content or clinical heterogeneity in terms of design and outcome, no meta-analysis was performed. The authors of this systematic review assessed clinical heterogeneity and felt that significant differences were present, reflecting the variation in patient characteristics in the clinical settings of the included reports. I^2^ was not calculated for statistical heterogeneity as no statistical comparison was performed. No adjustment was necessary due to clinical heterogeneity since the results were summarised in narrative expression.

### Certainty assessment

The certainty of evidence was determined using the GRADE working group methodology (www.gradeworkinggroup.org).^23^ Two authors (JS and RP) assessed the quality of evidence for the outcomes studied. Depending on the design of the included reports, the certainty of evidence was defined as high, moderate, low or very low. The certainty of evidence was then downgraded or upgraded based on different factors: certainty can be downgraded depending on individual report limitations (risk of bias), inconsistency of results, indirectness of evidence, imprecision or publication bias. In some cases, the certainty of evidence can be upgraded based on dose–response gradient, large magnitude of effects or addressed confounding. Finally, the certainty of evidence for the studied outcome was defined as high, moderate, low or very low.^23^ Since a meta-analysis was not performed, a qualitative approach for the five domains of GRADE was taken, as described by Murad *et al*.^24^

Risk of bias: a qualitative judgement is madeto assess the risk of bias across studies for an individual outcome. It is possible to consider the size of a study, its risk of bias and the impact it would have on the summarised effect.

Indirectness: a global qualitative judgement is made on how dissimilar the research evidence is to the clinical question at hand in terms of population, interventions and outcomes across studies.

Imprecision: a qualitative judgement is made by considering the optimal information size, that is, the total number of participants across all studies. Results may also be imprecise when the CIs of all the studies or of the largest studies include no effect and clinically meaningful benefits.

Inconsistency: a qualitative judgement of inconsistency is made by examining the consistency of effect direction and, most importantly, the disparity in effect sizes among studies. Considerable variation in effect estimates indicates inconsistency.

Publication bias: a qualitative judgement of publication bias can be made when the body of evidence consists of only small positive studies, or when studies are reported in trial registries but not published.

### Deviation from protocol

No deviations from the registered protocol were performed.

## Results

### Record selection

**Figure 1:**
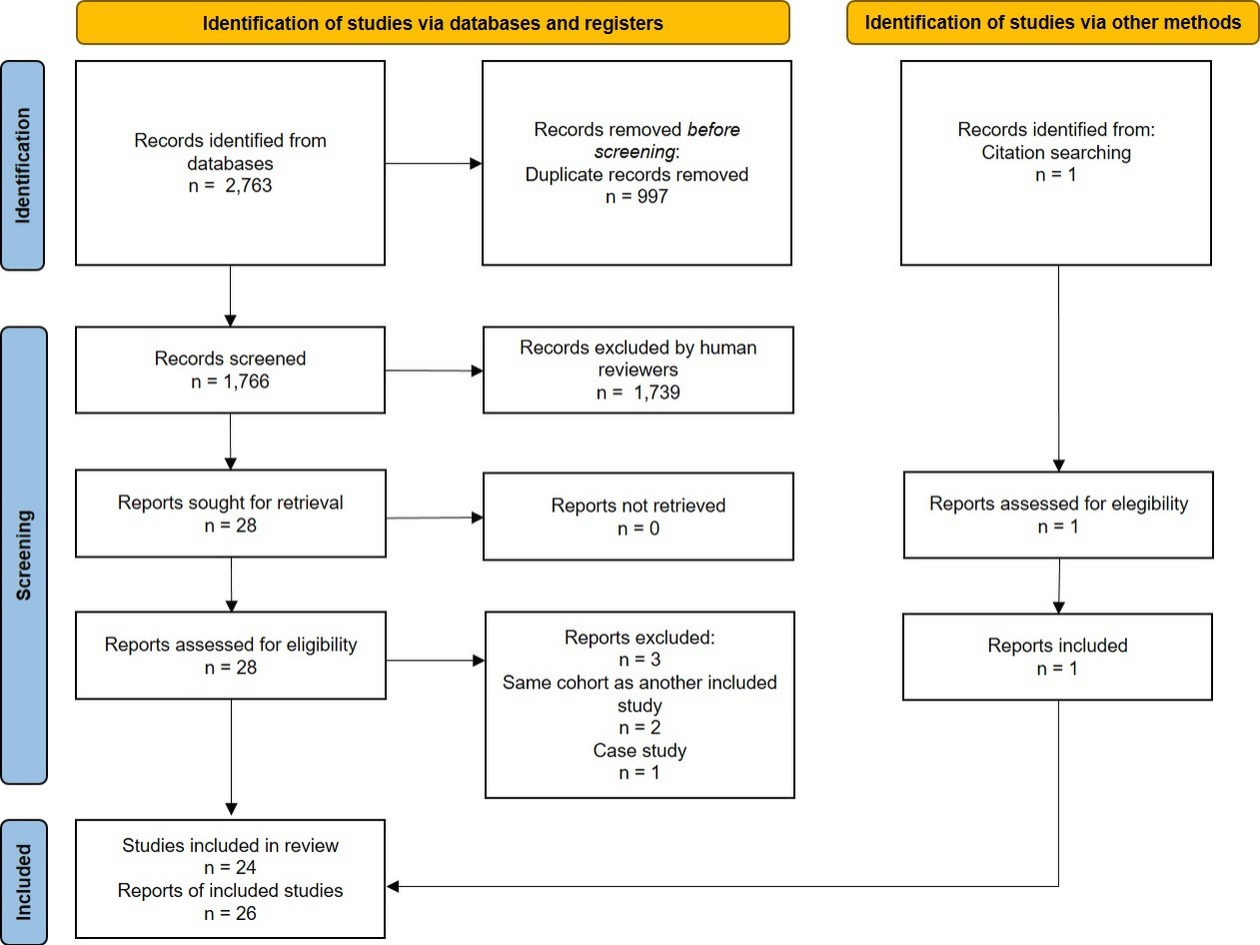
PRISMA flowchart of inclusion. PRISMA, Preferred Reporting Items for Systematic Reviews and Meta-Analyses.

The literature search yielded 2,763 records, of which 997 were duplicates. The screening process resulted in 28 reports being reviewed in full text, of which 26 reports from 24 studies were included. Reports excluded after full-text review and reasons are provided in online supplemental table 4. One additional report^25^ was identified from the screening of the reference list of included reports and systematic reviews of the topic of this review. This additional report was the oldest in our search results (published in 1974), which might be why the index search did not find it. Thus, 26 reports were finally included. The PRISMA flowchart shows the selection process in detail (figure 1). No new reports were identified from the updated search performed in 2021 and 2022. From the updated search performed in 2023, one new report^26^ was included.

### Report characteristics

The details of the included reports are presented in table 1. Outcomes for a total of 1,912 patients were extracted. For the reports which specified the type of MCL injury (I, II or III), outcomes for patients who suffered type I (n=215), type II (n=335) and type III (n=201) injuries were extracted. Table 1 shows the details of included studies, while online supplemental table 6 reports details of treatment and assessment stratified according to injury severity (grade I, II or III) for the reports reporting injury severity.

### Risk of bias

The risk of bias assessment performed with the RoBANS for each included report is presented in online supplemental table 5. No included report was at low risk of bias. A domain-based risk of bias summary is presented in table 2. Most reports were rated as having concerns in many risks of bias domains, with high or uncertain risk of bias in the selection of participants (73% of reports), confounding variables (69% of reports), measurement of exposure (65% of reports) and blinding of outcome assessment (92% of reports). Since both ‘selective outcome reporting’ and ‘incomplete outcome data’ were the two domains with the lowest percentage of reports at high risk of bias (1, respectively, 2 reports only), publication bias is unlikely to have affected results.

**Table 2:** Domain-based summary for the judgement of risk of bias

	Selection of participants	Confounding variables	Measurement of exposure	Blinding of outcome assessment	Incomplete outcome data	Selective outcome reporting
High risk of bias	19% (5/26)	11% (3/26)	30% (8/26)	27% (7/26)	8% (2/26)	4% (1/26)
Uncertain risk of bias	54% (14/26)	58% (15/26)	35% (9/26)	65% (13/26)	46% (12/26)	23% (6/26)
Low risk of bias	27% (7/26)	31% (8/26)	35% (9/26)	8% (2/26)	46% (12/26)	73% (19/26)

### Results of synthesis

#### Non-operative treatment

##### Exercise therapy

Five reports^25 27–30^ reported exercise therapy as an intervention with no brace or immobilisation. The grading of the evidence for exercise therapy was very low. Evidence started at level ‘low’ and was downgraded as follows:

- one point due to risk of bias, as no one of the five studies was as low risk of bias according to RoBANS.
- one point due to indirectness as there was a high degree of clinical heterogeneity in the type of training reported and the details of prescribed training.
- one point due to imprecision due to small sample size.

No downgrading was performed due to inconsistency or publication bias. Exercise description varied between isometric exercises and whirlpool treatments; low-speed running; full-speed running straight ahead, followed by figure-of-eight running and cutting; functional training programme involving progressive ROM and strength training followed by sport-specific; early functional rehabilitation; strengthening exercises, graded physiotherapy and sport-specific drills. Of 25 included reports, 10 reported on the type of training, with no details on dosage or specific exercises.^11 25 28 31–38^ In patients treated with training and no brace or immobilisation, the time to return to work was 1 week, the average lay-off time was slightly more than 3 weeks in professional elite male football players, while RTS time ranged from 3 to 12 weeks. The success rate (patients reporting excellent results on PROs, performance, or clinical assessment) varied between 70–98%. The average reported number of days missed from training practice for grade I injuries was 10, which increased to 35 days for grade II.

##### Knee brace

Thirteen reports described outcomes for patients treated with different types of knee brace.^11 27 31 32 35–37 39–44^ The evidence for the effect of using a knee brace in patients with knees that suffered an MCL injury was assessed according to GRADE as being of very low quality. Evidence started at level “low” and was then downgraded as follows:

- one point due to risk of bias, as the risk of bias according to RoBANS in the thirteen studies was uncertain at best;
- one point due to indirectness as there was a substantial clinical heterogeneity relating to the type of brace used for stabilisation, the length of time with a brace, injury severity (grade I, II or III) and rehabilitation performed during the time with a knee brace.
- one point due to imprecision due to small sample size.

No downgrading was performed due to inconsistency or publication bias. The time with a knee brace varied from two to 5 weeks. Three reports did not describe the time in a knee brace, as knee braces were applied until medial knee stress (assessed with a valgus stress test in 30° of knee flexion) produced no symptoms.^31 35 44^ All patients treated with knee braces were allowed to perform restricted leg training with different combinations of whirlpool, isotonic, isometric or isokinetic exercises.

More flexible knee braces produced a shorter lay-off time and better subjective outcomes than rigid braces.^27^ The RTS time varied between 4 and 9 weeks, but grade II injuries treated with a brace had a significantly longer lay-off time than grade II injuries not treated with a brace.^8^ All patients, irrespective of treatment, were reported to RTS. Clinical results were reported to be good to excellent in PROs, strength test assessments and X-rays 4 to 8 years after injury. However, grade III injuries showed significantly inferior results in each assessed clinical outcome.^43^

In 4 reports, the patients’ affected knees were completely immobilised.^11 32 39 40^ A decrease in the number of muscle fibres and sizes was observed in the quadriceps in the immobilised leg. Up to 2 years after injury, there was no significant difference in valgus stability, assessed with a valgus stress test at 30° of knee flexion or an OSI laxity tester (Orthopedic Systems Inc., Hayward, California) at 20° of knee flexion using a force of 90 Newton. No difference in the total score on PROs was observed between groups, and the results at the group level in the clinical examination were good or excellent.

##### Comparison between surgical and non-surgical treatment

The evidence for treatment outcomes between the surgical and non-surgical treatment of MCL injury was graded according to GRADE as being of very low quality. Evidence started as level “low” and was then downgraded as follows:

- one point due to risk of bias, as the risk of bias according to RoBANS was at best uncertain.
- one point due to indirectness as there was a substantial clinical heterogeneity in the type of surgery, assessment method and injury severity (grade I, II or III).
- one point due to imprecision due to small sample size.

No downgrading was performed due to inconsistency or publication bias. A larger decrease in muscle fibres and muscle fibre size and strength was reported in the affected leg of patients treated with surgery. However, no information regarding possible concomitant injuries was reported, and no matching between treatment groups was performed.^39 40^ There was no significant difference in valgus stability (assessed with a valgus stress test at 30° of knee flexion), or a total Marshall score attained with either the non-operative or the operative treatment of isolated MCL grade II or grade III injuries.^11 32^ In these reports,^11 32^ no matching was performed, but only isolated MCL injuries were compared across treatment groups, and in one report,^32^ cases were clinically comparable between treatment groups. In the same report, patients treated non-surgically regained strength significantly faster than patients treated with surgery.^32^ Furthermore, a greater proportion of success rate (ie, return to pre-injury level of performance) was reported in non-surgically treated isolated MCL injuries 6 weeks after injury/surgery (98% compared with 74% in the surgical group).^25^ No matching was performed, and data for multi-ligamentous injury was excluded from the analysis.^25^ Three reports described no difference in any clinical outcome, RTS rates or performance two or 10 years after MCL injury/surgery.^32 38 45^ Two of the reports^32 38^ included only isolated MCL injuries, but the report from Logan *et al*,^45^ did not separate knees with isolated MCL injuries from knees with MCL and other injuries. However, other injuries were defined as “prior” when MRIs for the actual MCL injury were screened.

##### Other treatments

Jones *et al* 34 reviewed patients with chronic pain after grade I and II MCL injuries treated with a corticosteroid injection, regardless of injury grading. Patients experienced immediate symptom resolution, and 80% were able to experience RTS after the injection. Yagishita *et al* 46 administered a hyperbaric oxygen therapy (HOT) protocol during five sessions within 10 days of injury, alongside rehabilitation: cryotherapy, compression and elevation until patients’ strength, proprioception and agility had recovered and they noticed that pain after therapy decreased significantly. Furthermore, the mean RTS time was 31 days in the HOT group and 41 in the control group. Zou *et al* 47 treated patients with a 3 month history of pain after MCL injury with three platelet-rich plasma (PRP) injections. They noticed a direct improvement in the International Knee Documentation Committee (IKDC) and Visual Analogue Scale (VAS). However, apart from the improvement directly after injection, no further improvement was noted up to the 6 month follow-up. Electrotherapy (10 and 50 Hz, respectively) was applied during the immobilisation period, but it revealed no significant differences in muscle cross-sectional area or isokinetic strength.^39 40^ Jang *et al* 26 treated 26 patients with grade III MCL injury with one atelocollagen injection 10 days after injury and compared outcomes with 24 patients without injection up to 12 months after injury. Results show less pain in the injection group at 6 and 12 months, but results are far from clinically relevant (eg, 0.4 vs 0.8 on a VAS at 12 months). Although, at 12 months, 80% of patients in the injection group returned to sport, compared with 50% in the non-injection group.^26^ No other treatments, such as laser or manual therapy, were reported in the included reports.

##### Summary of results

Of the 25 included reports, 18 were published before 2000 and 7 between 2009 and 2020. This suggests that the evidence relating to the non-surgical treatment of MCL injuries might need to be updated. Moreover, no difference was made between grade I and II injuries, where weight bearing and ambulation are tolerated, and muscle strengthening and range of motion (ROM) exercises are performed ‘in a standard fashion’.^46^ For grade III injuries, partial weight bearing has been recommended up to 4 weeks after injury. The overall description of the quality of rehabilitation protocols is very low, and details of exercise therapy have not been given. Substantial clinical heterogeneity was found in bracing, with reports describing bracing an MCL injury regardless of severity and other reports not applying a brace regardless of injury severity. The bracing time ranged between 1 and 10 weeks.

##### Certainty of evidence

The overall certainty of evidence based on GRADE was deemed ‘very low’ due to study design, indirectness of evidence and imprecision. Upgrading was not possible, which limits the certainty of results.

##### Quality of included reports

Overall, there was a fair quality of evidence for non-comparative reports assessed with the MINORS scale. The ratings for non-comparative reports ranged between 0 and 10 points, with a median of 9 points. Comparative reports had a range of 9–16 points, with a median of 13 points, thereby of fair quality (table 3). The most frequent methodological weakness was the need for a prospective sample size calculation and an unbiased assessment of the study endpoint. Methodological strengths were clearly stated, and the aims and endpoints were appropriate to the aim of the reports. Notably, no report was determined to be of high quality, with 10 reports being low or very low quality and 16 reports being of fair quality.

**Table 3:** Quality assessment using the Methodological Index for Non-Randomised Studies (MINORS)

Lead author	Items												Total score	Quality
	1	2	3	4	5	6	7	8	9	10	11	12		
Ballmer and Jakob^27^	2	0	1	1	0	2	0	0	1	2	0	0	9	Low
Derscheid and Garrick^31^	0	1	2	0	0	0	1	0					4	Very low
Ellsasser *et al* 25	0	0	0	0	0	0	0	0					0	Very low
Fetto and Marshall^11^	2	1	2	1	0	2	1	1	2	0	0	0	12	Low
Halkjear-Kristensen and Ingemann-Hansen^39^	2	1	2	2	0	2	2	0	2	2	0	1	16	Fair
Halkjear-Kristensen and Ingemann-Hansen^40^	0	1	2	1	0	2	2	0	2	2	0	1	13	Fair
Holden *et al* 28	0	2	1	0	0	0	2	0					5	Low
Indelicato^32^	2	2	2	0	0	2	1	0					9	Fair
Indelicato *et al* 33	2	2	2	2	0	2	1	0	2	1	0	0	14	Fair
Jang and Kim^26^	2	0	0	2	0	2	2	0	1	2	2	2	15	Fair
Jones *et al* 34	2	1	2	0	0	2	2	0					9	Fair
Jones *et al* 35	0	0	1	0	0	1	1	0					3	Very low
Kannus^41^	2	0	1	2	0	2	1	0					8	Fair
Murad *et al* 24	1	1	2	1	0	1	1	0	1	2	0	1	11	Low
Kannus^43^	2	1	1	2	0	2	2	0					10	Fair
Logan *et al* 45	2	2	0	1	0	2	2	0	2	2	0	1	14	Fair
Lundberg and Messner^29^	1	2	1	1	0	2	2	0					9	Fair
Lundberg and Messner^48^	2	0	1	2	0	2	2	0	1	0	2	1	13	Fair
Lundblad *et al* 44	2	0	1	1	0	2	0	0					6	Low
Lundblad *et al* 8	2	0	1	2	0	2	0	0					7	Low
Motamedi *et al* 30	2	2	1	2	0	2	0	0					9	Fair
Petermann *et al* 36	2	1	1	1	0	2	2	0					9	Fair
Reider *et al* 37	2	0	1	1	0	2	1	0					7	Low
Sandberg *et al* 38	2	2	1	2	0	2	0	0					9	Fair
Yagishita *et al* 46	2	1	1	2	0	2	1	0					9	Fair
Zou *et al* 47	1	2	1	1	0	2	2	0					9	Fair

## Discussion

This study systematically reviewed the current literature regarding the non-surgical treatment of isolated MCL injuries. A secondary objective was to investigate surgical and non-surgical treatment comparisons for MCL injuries. The main finding was that no differences in non-operative treatment had been reported between grade I and II injuries, where immediate weight bearing and ambulation were tolerated, and rehabilitation comprised different types of strengthening exercises with poorly reported details. For grade III MCL injuries, weight bearing was restricted up to 4 weeks after injury. Some reports^11 27 31 32 35–37 39–44^ used the immobilisation of the knee with a brace as a treatment method, while others did not use any equipment. The use of a brace was inconsistently reported, regardless of injury severity, and the duration of brace use varied substantially.

There was notable heterogeneity in the assessed outcomes and the choice of non-operative treatment, suggesting that knowledge of the non-operative treatment of isolated MCL injuries is scarce. Moreover, treatment protocols were poorly described and varied considerably, with publications reporting the same protocols for MCL injuries of all types, regardless of grading^25 28 29 31–34 36 39 40 43^ or reports describing different protocols for different injury grading.^8 11 30 42^ A similar pattern with great variety was observed for weight bearing, with reports reporting restrictions in weight bearing, regardless of injury severity^25 27 32 33 42 47^ and reports reporting no restriction in weight bearing, irrespective of injury severity.^11 28 29 31 35–40 46^ The protocols for bracing varied as well: some reports described bracing all injuries, regardless of injury severity^29 31–33 36 38–40^ other reports reported no bracing, irrespective of injury grade,^25 28 34 44 45 47 48^ while other reports reported bracing for different periods depending on injury severity.^8 11 43^ The brace time varied between 1 and 10 weeks. Some reports report a fixed time (eg, 2 or 4 weeks),^11 26 27 32 33 36–38 41 43^ while some reports report using a brace until symptom resolution^31 35^ and other reports did not describe the length of bracing,^8 29 46^ leaving clinicians with no knowledge of how to brace patients after isolated MCL injuries. As the practice of bracing after an MCL injury differs, there is an evident need for future research to determine when and how bracing should facilitate recovery from an MCL injury.

There was no guidance regarding when non-operative treatment was indicated or when surgery was needed. The authors suggest that the criteria mentioned in the introduction would be appropriate but admit that this is not ‘evidence based’.

Notably, most of the included reports (75%) were published before 1998. After that, there was a temporal gap until 2009, with no included reports. In the eight included reports produced between 2009 and 2023, four reported incidences of injuries from sports leagues with few details on treatment,^8 30 44 45^ two the effect of PRP or hyperbaric oxygen on pain,^46 47^ one the effect of an atelocollagen injection,^26^ and one the effect of steroid injections.^34^ In other words, since 1998, no or few publications have studied the effects and outcomes of different non-operative treatment regimens for isolated MCL injuries. Clinicians must, therefore, rely on knowledge that is now more than two decades old and likely suboptimal for the non-surgical treatment of isolated MCL injuries. In addition, this knowledge is substantially heterogeneous and needs to be better described. Consequently, clinicians need help finding guidance in the literature on how to treat isolated MCL injuries non-surgically. Therefore, it is time for researchers to produce high-quality studies assessing the effectiveness of different non-operative treatment regimens on outcomes after an isolated MCL injury.

### Limitations

Several limitations need to be acknowledged when interpreting the results of the present study, both regarding the evidence and the review process.

Concerning the evidence, there was substantial clinical heterogeneity in the included populations, the treatment methods, the length of treatment and the outcomes reported in the included reports. All ROBANS domains except ‘selective outcome reporting’ and ‘incomplete outcome data’ had most reports having a high or unclear risk of bias. The included reports were mostly rated as being of low or fair quality, according to MINORS. Thus, evidence in the included reports is more subject to a possible risk of bias. Consequently, the overall GRADE was rated as low, so the results should be interpreted with caution.

As for the review process, a limitation is the inclusion of English and Swedish languages only, even though most scientific publications are published in English. The treatment details provided in the included reports, such as exercises, volume, effort, progression benchmarks or regression in the event of symptoms, were very poor and sometimes not reported at all. Thus, performing a qualitative analysis was challenging, and therefore, further trials are needed before conclusions can be made about clinical practice.

## Conclusion

There is an evident knowledge gap in the non-operative treatment of isolated MCL injuries. This should prompt researchers and clinicians to produce high-quality evidence studies of the promising non-operative treatment of isolated MCL injuries to aid in decision-making and guide rehabilitation after MCL injury.

## Data Availability

Data are available from the corresponding author upon reasonable request.
