# Supplementary material for: Shedding light on the non-operative treatment of the forgotten side of the knee: rehabilitation of medial collateral ligament injuries—a systematic review
Source: BMJ Open Sport Exerc Med. 2024 Jun 25;10(2):e001750. doi: 10.1136/bmjsem-2023-001750 (PMC11202733; doi:10.1136/bmjsem-2023-001750)
Supplement: Supplementary data [file bmjsem-2023-001750supp001.pdf]

Appendix for systematic review: Shedding light on the non-operative treatment of the forgotten side of the knee: rehabilitation of medial collateral ligament injuries: a systematic review

Supplemental data 1-2: search results

Database: Embase
Date: 2020-11-11
No of results: 1229 ref

Database(s): **Embase** 1974 to 2020 November 10
Search Strategy:

Supplemental data Table 1: Search results for the search on Embase

#	Searches	Results
1	exp knee medial collateral ligament/	240
2	Tibial Collateral Ligament.ab,kw,ti.	48
3	knee medial collateral ligament.ab,kw,ti.	26
4	Medial Collateral Ligament.ab,kw,ti.	2647
5	knee.ab,kw,ti.	188537
6	4 and 5	1668
7	1 or 2 or 6	1805
8	animals/ not (animals/ and humans/)	938339
9	(animal or animals or rat or rats or mouse or mice or rodent or rodents or dog or dogs or cat or cats or hamster or hamsters or rabbit or rabbits or swine or murine).ti.	2024806
10	8 or 9	2753946
11	7 not 10	1600
12	(child not (child and adult)).sh.	1199360
13	(Child or children or pediatric* or paediatric* or infant*).ti.	1121142
14	12 or 13	1730665
15	11 not 14	1569
16	limit 15 to (danish or english or norwegian or swedish)	1393
17	limit 16 to (books or chapter or conference abstract or "conference review" or editorial or erratum or letter or short survey or tombstone)	164
**18**	**16 not 17**	**1229**

Database: MEDLINE
Date: 2020-11-11
No of results: 1352 ref

Supplemental data Table 2: Search results for the search on MEDLINE

Search	Query	Results
#22	Search: **#18 NOT #21** Filters: **Danish, English, Norwegian, Swedish** Sort by: **Most Recent**	1,352
#21	Search: **#19 OR #20** Filters: **Danish, English, Norwegian, Swedish** Sort by: **Most Recent**	1,311,063
#20	Search: **Child[ti] OR children[ti] OR pediatric*[ti] OR paediatric*[ti] OR infant*[ti]** Filters: **Danish, English, Norwegian, Swedish** Sort by: **Most Recent**	801,099
#19	Search: **((child[mh]) NOT (child[mh] AND adult[mh]))** Filters: **Danish, English,**	962,611

	**Norwegian, Swedish** Sort by: **Most Recent**	
#18	Search: **#13 NOT #17** Filters: **Danish, English, Norwegian, Swedish** Sort by: **Most Recent**	1,375
#17	Search: **#14 OR #16** Filters: **Danish, English, Norwegian, Swedish** Sort by: **Most Recent**	4,623,379
#16	Search: **animal[ti] OR animals[ti] OR rat[ti] OR rats[ti] OR mouse[ti] OR mice[ti] OR rodent[ti] OR rodents[ti] OR dog[ti] OR dogs[ti] OR cat[ti] OR cats[ti] OR koalas[ti] OR hamster[ti] OR hamsters[ti] OR rabbit[ti] OR rabbits[ti] OR swine[ti] OR murine[ti]** Filters: **Danish, English, Norwegian, Swedish** Sort by: **Most Recent**	1,707,757
#14	Search: **((animals[mh]) NOT (animals[mh] AND humans[mh]))** Filters: **Danish, English, Norwegian, Swedish** Sort by: **Most Recent**	4,300,436
#13	Search: **#11 NOT #12** Filters: **Danish, English, Norwegian, Swedish** Sort by: **Most Recent**	1,735
#12	Search: **(Editorial[ptyp] OR Letter[ptyp] OR Comment[ptyp])** Filters: **Danish, English, Norwegian, Swedish** Sort by: **Most Recent**	1,775,826
#11	Search: **#1 OR #2 OR #3 OR #6** Filters: **Danish, English, Norwegian, Swedish** Sort by: **Most Recent**	1,754
#10	Search: **#1 OR #2 OR #3 OR #6** Filters: **Danish, English, Norwegian** Sort by: **Most Recent**	1,754
#9	Search: **#1 OR #2 OR #3 OR #6** Filters: **Danish, English** Sort by: **Most Recent**	1,753
#8	Search: **#1 OR #2 OR #3 OR #6** Filters: **Danish** Sort by: **Most Recent**	4
#7	Search: **#1 OR #2 OR #3 OR #6** Sort by: **Most Recent**	1,926
#6	Search: **#4 AND #5** Sort by: **Most Recent**	1,367
#5	Search: **knee[tiab]** Sort by: **Most Recent**	145,919
#4	Search: **Medial Collateral Ligament[tiab]** Sort by: **Most Recent**	2,222
#3	Search: **knee medial collateral ligament[tiab]** Sort by: **Most Recent**	27
#2	Search: **Tibial Collateral Ligament[tiab]** Sort by: **Most Recent**	48
#1	Search: **knee medial collateral ligament[mesh]** Sort by: **Most Recent**	970

Database: Pedro
Date: 2020-11-11
No of results: 10 ref

Supplemental data Table 3; Overview of hits in different databases

**Database**	**Record**
Embase	1,229
MEDLINE	1,352
PEDro	10
Total	2,591 in November 2020 + 172 in December 2021 + 246 in November 2022 and + 204 in January 2024.
Total unique records	1,969

Embase: Excerpta Medica dataBASE, MEDLINE: Medical Literature Analysis and Retrieval System Online, PEDro: Physiotherapy Evidence

Database

Supplemental data Table 4: reports excluded at full-text review and reason

Report excluded	Reason
Lundberg M, Messner K. Decrease in valgus stiffness after medial knee ligament injury. A 4-year clinical and mechanical follow-up study in 38 patients. Acta Orthop Scand. 1994;65(6):615-9	Same cohort as the 10 years follow-up, included in the Review (35)
Kannus, P.; Jarvinen, M.; Strength of quadriceps and hams lateral collateral ligament insufficiency. Scandinavian Journal of Sports Sciences - Volume 9, Issue 3, pp. 79-83 - published 1987-01-01	Same cohort as the Hamstring/strength quadriceps ratio Included in the review (32)
Acevedo J, Boden AL, Greif DN, Emerson CP, Ruiz JT, Jose J, Feigenbaum LA, Kaplan LD. Distal Medial Collateral Ligament Grade III Injuries in Collegiate Football Players: Operative Management, Rehabilitation, and Return to Play. J Athl Train. 2021 Jun 1;56(6):565-571.	Case study

Supplemental data Table 5: risk of bias

Supplemental data Table 5: Risk of bias rating in included studies with Risk of Bias Assessment tool for Non-randomized Studies (RoBANS).

**Lead author**	Selection of participants	Confounding variables	Measurement of exposure	Blinding of outcome assessment	Incomplete outcome data	Selective outcome reporting
Ballmer et al.(1)	+	−	+	×	+	−
Derscheid et al.(2)	×	−	×	×	−	−
Ellsasser et al.(3)	×	−	×	−	−	−
Fetto et al.(4)	×	−	×	×	×	−
Halkjear et al.(5)	−	×	×	−	−	−
Halkjear et al.(6)	−	×	×	−	−	−
Holden et al.(7)	−	×	+	−	−	×
Indelicato et al.(8)	+	−	−	×	+	+
Indelicato et al.(9)	+	−	+	−	+	+
Jang et al.(10)	−	+	+	+	×	+
Jones et al.(11)	−	+	+	−	+	+
Jones et al.(12)	−	+	−	−	+	+
Kannus et al.(13)	×	−	−	×	+	+
Kannus et al.(14)	−	−	−	×	−	+
Kannus et al.(15)	−	−	−	×	−	+
Logan et al.(16)	+	−	×	+	+	+
Lundberg et al.(17)	+	−	+	−	+	+
Lundberg et al.(18)	−	+	+	−	+	+
Lundblad et al.(19)	−	+	×	−	−	+
Lundblad et al.(20)	−	+	−	−	−	+
Motamedi et al.(21)	×	−	+	−	+	+
Petermann et al.(22)	−	−	×	−	−	+
Reider et al.(23)	−	+	−	−	−	+
Sandberg et al.(24)	+	+	−	−	−	+
Yagishita et al.(25)	−	−	−	−	+	+
Zou et al.(26)	+	−	+	−	+	+

+ = low risk of bias; × = high risk of bias; − = uncertain risk of bias.

Supplemental data Table 6: Details of assessment, type and length of bracing and type and length of rehabilitation in included studies.

Author	Injury severity	Assessment method	Type of bracing	Length of bracing	Rehabilitation details	Length of rehabilitation
Ballmer et al.(1)	Only grade III injuries	Valgus stress test in 30° of flexion, radiographics and arthroscopy	Elastic wrap (group 1)<br>or<br>plaster cast and elastic wrap (group 2)	8 weeks<br><br>4+4 weeks	Partial weight-bearing	4 weeks
Derscheid et al.(2)	Same protocol regardless of injury severity	Valgus stress test in 30° of flexion and full extension, tenderness, radiographics	Compression with elastic wrap and knee immobiliser at 10-15° of flexion	Until more than 90° of painless range of motion was achieved	Whirlpool, isotonic, isokinetic exercises and sport-specific drills	Until return to full participation
Ellsasser et al.(3)	Injury severity not reported	Valgus stress test in 30° of flexion and full extension. Local tenderness and radiographics	No bracing used	n.a.	No weight-bearing<br>Isometric and whirlpool exercises<br>Quadriceps, hamstring and gastrocnemius exercises	1 week<br><br>Until RTS (3-8 weeks)
Fetto et al.(4)	I<br>II<br><br>III	Physical and radiographic tests	Knee splint<br>Cylinder plaster<br><br>Cylinder plaster	2 weeks<br>4-6 weeks<br><br>6-10 weeks	Muscle strengthening programme for quadriceps and hamstrings. No difference depending on injury severity	n.r.
Halkjear et al.(5, 6)	Injury severity not reported	Valgus stress test in 15° of flexion	Immobilisation in plaster of Paris	Mean 31 days (29-41 days)	Five groups with a) no exercise; b) isometrics; c) and d) electric stimulation; e) bicycle on uninjured leg only	
Holden et al.(7)	Same protocol regardless of injury severity (I-II)	Valgus stress test in 30° of flexion and full extension, tenderness, radiographic examination	Not used	n.a.	First week: ROM + eccentric; second week: endurance, walking, whirlpool exercise; third week: running, resistance exercises, agility drills; fourth week: RTS	3-4 weeks
Indelicato et al.(8)	Same protocol regardless of injury severity	Valgus stress test (under anaesthesia); arthroscopic examination	Toe-groin plaster with knee in varus and 30° of flexion<br><br>Rigid cast first, then ankle to groin fibreglass cast brace (knee motion 30-80° flexion) at 2 weeks	6 weeks for surgical repair group<br><br>2+4 weeks for non-surgical group	Active and active-assisted ROM exercises. Crutch walking to 90° knee flexion. Isometric isotonic and isokinetic exercises	n.r.
Indelicato et al.(9)	Same protocol regardless of injury severity	Valgus stress test (under anaesthesia) and arthroscopic examination	Long leg cast with 30° knee flexion.<br><br>Long leg orthosis 30-90° knee flexion	2 weeks<br><br>4 weeks	Weight-bearing with crutches when tolerated. Isometric isotonic and isokinetic exercises. Running within two weeks of cast removal	Until complete recovery of strength, power and endurance of opposite leg
Jang et al.(10)	Only grade III injuries	MRI findings	Hinged knee brace	4 weeks after 2 weeks of immobilisation	Weight bearing if pain was tolerable	Up to 3 months

Jones et al.(11)	Same protocol regardless of injury severity (I-II)	Thickened tissue of intermediate signal on MRI or slightly echogenic on ultrasound consistent with solid scar tissue in the region of the deep MCL with a history of three months of medial knee pain	Not used	n.a.	n.r.	n.r.
Jones et al.(12)	Only grade III injuries	4+⁑ coronal plane instability with knee in flexion, negative sagittal plane stress and lack of instability with knee in extension	Off-the-shelf knee brace with coronal plane correction strap	1 week Increase brace ROM weekly	Straight leg raises and isometric exercises immediately. Quadriceps and hamstring exercises with springs applied to knee brace. Running began when brace was removed.	Until full ROM, strength and running and cutting ability returned to normal (34 days after injury)
Kannus et al.(13, 14) †	Same protocol regardless of injury severity	Arthroscopy and valgus stress test in 30° of flexion	n.r.	n.r.	n.r.	n.r.
Kannus et al.(15)	I	None included				
	II	Valgus stress test in 30° of flexion and full extension	Plaster cast, posterior splint or knee bandage	3-6 weeks	Isometric quadriceps exercise during immobilisation. Weight-bearing after 2-4 weeks	At least six months under supervision
	III	Valgus stress test in 30° of flexion and full extension		4-9 weeks		
Logan et al.(16)	Injury severity not reported	MRI findings	n.r.	n.r.	n.r.	n.r.
Lundberg et al.(17)	Same protocol regardless of injury severity (I-II)	Arthroscopic evaluation	Functional bracing or elastic wrapping	n.r.	Weight-bearing and ambulation as soon as tolerated	n.r.
Lundberg et al.(18)	Injury severity not reported	Manual examination under anaesthesia and arthroscopic evaluation	n.r.	n.r.	Individual and functional rehabilitation programme	Until full recovery
Lundblad et al.(19)	Injury severity not reported	n.a.	n.r.	n.r.	n.r.	n.r.
Lundblad et al.(20)	I	Valgus stress test in semi-flexion and full extension, MRI	Stabilising knee brace (6.7% of patients)	n.r.	n.r.	10 days' lay-off time
	II		Stabilising knee brace (53.1% of patients)	n.r.	n.r.	n.r.
	III		Stabilising knee brace (100% of patients)	n.r.	n.r.	n.r.
Motamedi et al.(21)	I	History, physical examinations and knee radiographics	n.r.	n.r.	n.r.	Missed 10 days of practice
	II		n.r.	n.r.	n.r.	Missed 35 days of practice
	III		n.r.	n.r.	n.r.	n.r.
Petermann et al.(22)	Same protocol regardless of injury severity	Valgus stress test in 30° of flexion	Long leg fibreglass cast brace with metal hinges allowing 10-90° of flexion	4 weeks	Full weight-bearing as tolerated. Muscle strengthening and ROM exercises	Return to activity after eight weeks

Reider et al.(23)	Only grade III	Tenderness over MCL and abnormally increased valgus laxity with soft end point with knee in flexion	Lateral knee hinge brace with no ROM restriction.	3 weeks	Weight-bearing as tolerated. ROM exercises in whirlpool. Resistance exercise when 90° of flexion was achieved. Running when full ROM was achieved	Until full ROM, minimal pain, 90% symmetrical quadriceps strength and full running ability was restored
Sandberg et al.(24)	Injury severity not reported	Stability test under anaesthesia	Plaster cast with knee in 30° flexion.	6 weeks	Isometrics when in plaster. Exercise to improve dynamic strength	RTS when joint was free from symptoms and 85% LSI
Yagishita et al.(25)	Only grade II injuries	Manual examination (including valgus stress test and tenderness) and right-left asymmetries	A hinged knee brace when the athlete had moderate or severe pain.	No details provided	Muscle strengthening and ROM exercises in a standard fashion	Until muscle strength, proprioception, agility and cardiopulmonary function had recovered to levels comparable to contralateral side
Zou et al.(26)	Only grade I-II injuries	MRI	n.r.	n.r.	Range-of-motion exercises were started and weight-bearing was gradually introduced. Total weight-bearing was permitted if pain free.	n.r.

n.a. = not applicable; n.r. = not reported; RTS = Return To Sport; ROM = range of motion; LSI = Limb Symmetry Index; MRI = Magnetic Resonance Imaging; ‡ = unclear what 4+ coronal plane instability refers to. † = For the two studies by Kannus et al.(13, 14) and Halkjaer et al.,(5, 6) different outcomes for the same cohort were reported in different publications. As a result, both studies are included, but the number of patients was counted once.

1. Ballmer PM, Jakob RP. The non operative treatment of isolated complete tears of the medial collateral ligament of the knee. A prospective study. Arch Orthop Trauma Surg (1978). 1988;107(5):273-6.
2. Derscheid GL, Garrick JG. Medial collateral ligament injuries in football. Nonoperative management of grade I and grade II sprains. Am J Sports Med. 1981;9(6):365-8.
3. Ellsasser JC, Reynolds FC, Omohundro JR. The non-operative treatment of collateral ligament injuries of the knee in professional football players. An analysis of seventy-four injuries treated non-operatively and twenty-four injuries treated surgically. J Bone Joint Surg Am. 1974;56(6):1185-90.
4. Fetto JF, Marshall JL. Medial collateral ligament injuries of the knee: a rationale for treatment. Clin Orthop Relat Res. 1978(132):206-18.
5. Halkjaer-Kristensen J, Ingemann-Hansen T. Wasting of the human quadriceps muscle after knee ligament injuries, IV dynamic and static muscle function. Scand J Rehabil Med Suppl. 1985;13:29-37.
6. Halkjaer-Kristensen J, Ingemann-Hansen T. Wasting of the human quadriceps muscle after knee ligament injuries, II muscle fibres morphology. Scand J Rehabil Med Suppl. 1985;13:12-20.
7. Holden DL, Eggert AW, Butler JE. The nonoperative treatment of grade I and II medial collateral ligament injuries to the knee. Am J Sports Med. 1983;11(5):340-4.
8. Indelicato PA. Non-operative treatment of complete tears of the medial collateral ligament of the knee. J Bone Joint Surg Am. 1983;65(3):323-9.
9. Indelicato PA, Hermansdorfer J, Huegel M. Nonoperative management of complete tears of the medial collateral ligament of the knee in intercollegiate football players. Clin Orthop Relat Res. 1990(256):174-7.
10. Jang YH, Kim DS. Atelocollagen Injections Improve Outcomes in the Nonsurgical Treatment of Grade III Medial Collateral Ligament Injuries. Clin Orthop Surg. 2023;15(6):953-9.
11. Jones L, Bismil Q, Alyas F, Connell D, Bell J. Persistent symptoms following non operative management in low grade MCL injury of the knee - The role of the deep MCL. Knee. 2009;16(1):64-8.
12. Jones RE, Henley MB, Francis P. Nonoperative management of isolated grade III collateral ligament injury in high school football players. Clin Orthop Relat Res. 1986(213):137-40.
13. Kannus P. Relationship between peak torque and total work in an isokinetic contraction of the medial collateral ligament insufficient knee. Int J Sports Med. 1988;9(4):294-6.

14. Kannus P. Hamstring/quadriceps strength ratios in knees with medial collateral ligament insufficiency. Isokinetic and isometric results and their relation to patients' long-term recovery. J Sports Med Phys Fitness. 1989;29(2):194-8.
15. Kannus P. Long-term results of conservatively treated medial collateral ligament injuries of the knee joint. Clin Orthop Relat Res. 1988(226):103-12.
16. Logan CA, Murphy CP, Sanchez A, Dornan GJ, Whalen JM, Price MD, et al. Medial Collateral Ligament Injuries Identified at the National Football League Scouting Combine: Assessment of Epidemiological Characteristics, Imaging Findings, and Initial Career Performance. Orthop J Sports Med. 2018;6(7):2325967118787182.
17. Lundberg M, Messner K. Long-term prognosis of isolated partial medial collateral ligament ruptures. A ten-year clinical and radiographic evaluation of a prospectively observed group of patients. Am J Sports Med. 1996;24(2):160-3.
18. Lundberg M, Messner K. Ten-year prognosis of isolated and combined medial collateral ligament ruptures. A matched comparison in 40 patients using clinical and radiographic evaluations. Am J Sports Med. 1997;25(1):2-6.
19. Lundblad M, Waldén M, Magnusson H, Karlsson J, Ekstrand J. The UEFA injury study: 11-year data concerning 346 MCL injuries and time to return to play. Br J Sports Med. 2013;47(12):759-62.
20. Lundblad M, Hägglund M, Thomeé C, Hamrin Senorski E, Ekstrand J, Karlsson J, Waldén M. Medial collateral ligament injuries of the knee in male professional football players: a prospective three-season study of 130 cases from the UEFA Elite Club Injury Study. Knee Surg Sports Traumatol Arthrosc. 2019;27(11):3692-8.
21. Motamedi AR, Gowd AK, Nazemi AK, Gardner ST, Behrend CJ. Incidence, Positional Distribution, Severity, and Time Missed in Medial Collateral Ligament Injuries of the Knee in NCAA Division I Football Athletes. J Am Acad Orthop Surg Glob Res Rev. 2017;1(5):e019.
22. Petermann J, von Garrel T, Gotzen L. Non-operative treatment of acute medial collateral ligament lesions of the knee joint. Knee Surg Sports Traumatol Arthrosc. 1993;1(2):93-6.
23. Reider B, Sathy MR, Talkington J, Blyznak N, Kollias S. Treatment of isolated medial collateral ligament injuries in athletes with early functional rehabilitation. A five-year follow-up study. Am J Sports Med. 1994;22(4):470-7.
24. Sandberg R, Balkfors B, Nilsson B, Westlin N. Operative versus non-operative treatment of recent injuries to the ligaments of the knee. A prospective randomized study. J Bone Joint Surg Am. 1987;69(8):1120-6.
25. Yagishita K, Enomoto M, Takazawa Y, Fukuda J, Koga H. Effects of hyperbaric oxygen therapy on recovery acceleration in Japanese professional or semi-professional rugby players with grade 2 medial collateral ligament injury of the knee: A comparative non-randomized study. Undersea Hyperb Med. 2019;46(5):647-54.
26. Zou G, Zheng M, Chen W, He X, Cang D. Autologous platelet-rich plasma therapy for refractory pain after low-grade medial collateral ligament injury. J Int Med Res. 2020;48(2):300060520903636.
